# PARP-1 inhibition influences the oxidative stress response of the human lens

**DOI:** 10.1016/j.redox.2016.03.003

**Published:** 2016-03-07

**Authors:** Andrew J.O. Smith, Simon S.R. Ball, Richard P. Bowater, I. Michael Wormstone

**Affiliations:** School of Biological Sciences, University of East Anglia, Norwich Research Park, Norwich NR4 7TJ, UK

**Keywords:** Poly (ADP-ribose) polymerase-1, Lens, Cataract, DNA damage, Cell survival, Human

## Abstract

Poly(ADP-ribose) polymerase-1 (PARP-1) is best characterised for its involvement in DNA repair. PARP-1 activity is also linked to cell fate, confounding its roles in maintaining genome integrity. The current study assessed the functional roles of PARP-1 within human lens cells in response to oxidative stress. The human lens epithelial cell line FHL124 and whole human lens cultures were used as experimental systems. Hydrogen peroxide (H_2_O_2_) was employed to induce oxidative stress and cell death was assessed by LDH release. The functional influence of PARP-1 was assessed using targeted siRNA and chemical inhibition (by AG14361). Immunocytochemistry and western blotting were used to assess PARP-1 expression and the alkaline comet assay determined the levels of DNA strand breaks. PARP-1 was generally observed in the cell nucleus in both the FHL124 cell line and whole human lenses. PARP-1 inhibition rendered FHL124 cells more susceptible to H_2_O_2_-induced DNA strand breaks. Interestingly, reduction of PARP-1 activity significantly inhibited H_2_O_2_-induced cell death relative to control cells. Inhibition of PARP-1 in whole human lenses resulted in a reduced level of lens opacity and cell death following exposure to H_2_O_2_ relative to matched pair controls. Thus, we show that PARP-1 could play a role in the fate of human lens cells, and these first observations in human lenses suggest that it could impact on lens opacity. Further studies are required to elucidate the regulatory processes that give rise to these effects.

## Highlights

• PARP-1 is found in the FHL124 lens cell line and whole human lens.
• PARP-1 inhibition increases H_2_O_2_-induced DNA strand breaks in human lens cells.
• Suppression of PARP-1 counters H_2_O_2_-induced human lens cell death.
• Inhibition of PARP-1 reduces H_2_O_2_-induced human lens opacity.
• PARP-1 could play a role in the fate of human lens cells and lens opacity.

## Graphical abstract

**Figure:**
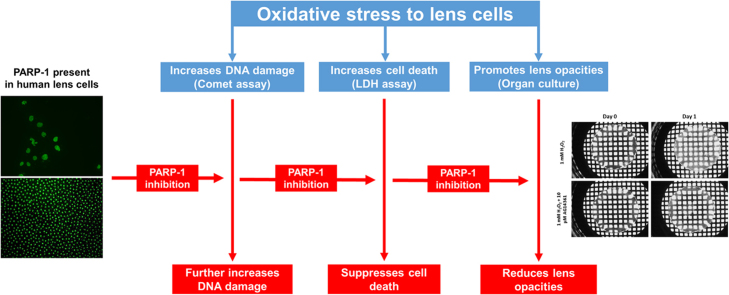

## 1 Introduction

Poly-ADP-ribose polymerases (PARPs) are a family of proteins that are linked with a wide-range of DNA repair pathways [1], [2], [3], [4], [5], [6]. Some PARPs have been identified as targets for therapeutics for human diseases, particularly cancers [7], [8], [9], [10]. However, a major complexity in developing such compounds is that PARP enzymes have multiple cellular roles, acting both in cell survival and in cell death pathways [5], [11]. In the current study, we used the human lens as an experimental model to evaluate the physiological functions of human PARP-1 in both cells and the organ.

The genomes of all cells are continually damaged in many ways, by many agents. These can be external to the cell, for example UV radiation from sunlight, and internal, such as chemicals generated by normal cellular metabolism [12], [13]. Such damage can disrupt normal cellular metabolism, leading to mutations and potentially cell death, and all cells have a complex of networks to detect and correct damage to DNA. For example: Nucleotide Excision Repair (NER) repairs bulky lesions such as pyrimidine dimers; Base Excision Repair (BER) repairs damage to bases; and Non-homologous End-joining (NHEJ) repairs double-strand breaks (DSBs) in the DNA backbone [12], [13], [14]. The human genome contains ~150 different genes that are devoted to DNA repair processes [15], [16], whose importance is underscored by the debilitating clinical symptoms that arise when these are defective [14]. Accumulated DNA damage is also associated with natural ageing phenomena [13], [17]. The significance of the relationship between DNA damage/repair and human diseases was recognised in 2015 by the award of the Nobel Prize in Chemistry to Tomas Lindahl, Aziz Sancar, and Paul Modrich [18]. Thus, a detailed understanding of DNA repair mechanisms will provide insights into biological processes that are fundamental to improving lifelong health and wellbeing in humans.

Through their roles in a wide-range of DNA repair pathways, the PARP family of proteins are central to genome integrity [3], [4], [19], [20], [21], [22], [23]. PARPs act to convert nicotinamide adenine dinucleotide (NAD^+^) to ADP-ribose, catalysing addition of polymers of ADP-ribose (PAR) to various nuclear acceptor proteins, including histone, p53 and PARPs themselves [5], [20], [24], [25]. Multiple PARPs have been identified that have a well conserved catalytic domain but have different structural domains and functions [3], [4], [20]. PARPs are divided into three groups: (i) PARPs 1–5, which are bona fide PARPs; (ii) PARPs 6–8, 10–12 and 14–16, which are confirmed or putative mono-ADP-ribosyl transferases; (iii) PARPs 9 and 13, which are likely to be inactive because they lack key NAD^+^-binding residues. PARP activities have been linked to diverse cellular processes, including the response to DNA damage, chromatin remodelling, transcriptional regulation, intracellular trafficking, telomere cohesion and energy metabolism [24], [25]. Among the PARP family members, PARPs 1–3 have been most widely studied and they have well defined roles in the DNA damage response, but the cellular roles of other family members are incompletely understood [5], [23]. These PARPs have been identified as targets for potential therapeutics for human diseases, including cancer [7], [8], [9], [10]. However, clinical trials have provided conflicting data, leading to re-evaluation of these therapies and highlighting that there is still much to learn about the physiological roles of these proteins in humans [5], [25], [26], [27].

Genotoxic stress leads to PARP activation and various cellular responses, including a decrease of levels of NAD^+^ and increase of levels of PAR. Through these biochemical activities the PARPs dramatically influence the energy balance of cells and this may provide a link to different physiological outcomes [3], [4], [5], [21], [23]. This has led to the proposal that PARP-1 (at least) may act as a “cellular rheostat” in relation to stress, promoting different cellular responses to various types and levels of stress signals [5]. Thus, as the strength of the stress stimulus increases, the levels of PARP-1 activity and PAR synthesis increase, leading to different cellular outcomes. Medium levels of PARP activity promote repair of DNA damage, returning cells to homeostasis and genome stability. When levels of DNA damage exceed repair capacity or there is prolonged PARP activity, programmed cell death (PCD) is activated to prevent cells from accumulating deleterious mutations. Apoptosis is a well-characterised mechanism of PCD that involves caspases and other well-studied proteins such as p53 [13], [14], [17]. Due to the activities of PARPs, extensive DNA damage is accompanied by large-scale PAR synthesis that can lead to a unique form of cell death, termed parthanatos. This triggers translocation of Apoptosis Inducing Factor (AIF) to the nucleus where it mediates large-scale DNA fragmentation and cell death [5], [23]. PAR polymers deliver different signals depending on their length and branching characteristics and, thus, PARP activity acts as an elegant switch between DNA repair and induction of PCD [5], [23]. There is growing evidence that ageing in humans leads to higher levels of DNA damage, which may be due to reduced effectiveness of DNA repair processes [13], [17], [28], [29]. Importantly, loss (or inhibition) of PARP activity may lead to increased levels of DNA damage and reduced amounts of PCD, including through parthanatos.

Although PARP function has been highly studied at an organismal level for some mammals, such as the mouse, the data is less strong in humans, partly because knowledge about the number and possible function of human PARPs is limited [3], [20], [23]. The unique biology of the lens and its anatomical situation provides a valuable human system to investigate PARP activity in relation to DNA repair capability and tissue physiology. The lens is isolated from other tissues and cells within the central anterior epithelium have been present since lens vesicle formation during embryogenesis. Moreover, both cell death and division are negligible in this population [30]. Consequently, for very long timeframes these cells are exposed to continuous stress, from UV exposure and generation of metabolic products, and this is likely to impose a significant burden of biomolecular stress as the individual ages. Indeed, recently we have used this system to demonstrate important links between oxidative stress, DNA repair systems and the health of the lens [28], [29]. We therefore employed a human lens epithelial cell line (FHL124) and whole human lens organ culture as experimental models to evaluate the physiological functions of human PARP-1. We provide the first confirmation that PARP-1 protein is expressed in the native human lens; in addition, expression in a lens cell line (FHL124) was also observed. Moreover, inhibition of PARP-1 protects FHL124 cells against H_2_O_2_-induced cell death whilst rendering them more susceptible to persistent DNA strand breaks. Most importantly, PARP-1 inhibition in whole human lenses protects them from oxidative stress-induced opacity and cell death, suggesting that this could provide a novel target for therapies to suppress lens opacity and cataract formation.

## 2 Materials and methods

### 2.1 Cell culture

The FHL124 human lens epithelial cell line is a non-virally transformed cell line generated from human capsule-epithelial explants [31]. This cell line demonstrates a 99.5% homology to native lens epithelium in transcript profile [32]. FHL124 cells were cultured at 35 °C with 5% CO_2_, 95% air in Eagle's Minimum Essential Medium (EMEM) (Gibco, Paisley, UK) supplemented with 5% v/v foetal calf serum (FCS) (Gibco) and 50 μg/ml gentamcin (Sigma-Aldrich, Dorset, UK). FHL124 cells were seeded onto 35 mm tissue culture dishes (35,000/dish for alkaline comet assay and western blot) or 18×18 class coverslips contained within 35 mm tissue culture dishes (10,000/coverslip for immunocytochemistry). The culture medium was replaced with EMEM without FCS 24 h prior to addition (or start) of experimental conditions.

### 2.2 Whole human lens culture

Donor eyes were obtained from the East Anglian Eye Bank with written informed consent obtained from next of kin. The research was approved by the UK National Research Ethics Committee (REC 04/Q0102/57) and followed the tenets of the Declaration of Helsinki regarding the use of human donor material. Following removal of cornea for transplant, lenses were dissected by anterior approach and cultured within 24 h post-mortem as previously described [33]. Briefly, following dissection, lenses were maintained in EMEM supplemented with 50 μg/ml gentamicin at 35 °C, 5% CO_2_, 95% air for 24 h prior to addition of experimental conditions to ensure no damage had arisen during dissection.

### 2.3 Chemical inhibition of PARP-1

PARP-1 was chemically inhibited with AG14361 (Selleck Chemicals, Houston, TX, USA), a potent and specific inhibitor of PARP-1 [34], [35]. AG14361 was diluted in DMSO (Fisher Scientific, Loughborough, UK) upon delivery to produce a 25 mM stock which was frozen at −80 °C. All experiments therefore also contained a dimethyl sulfoxide (DMSO) control diluted to the same concentration. AG14361 was freshly diluted in serum-free EMEM and added to FHL124 cells cultures to give a final concentration of 1 µM. For experiments using whole human lenses, freshly diluted AG14361 was added to whole human lens cultures to give a final concentration of 10 μM.

### 2.4 Knockdown of PARP-1 expression

Expression of PARP-1 was depleted in FHL124 cells using targeted siRNA (Qiagen, Crawley, UK) with controls transfected with AllStars Negative control siRNA (scramble siRNA) (Qiagen) using oligofectamine transfection reagent (Invitrogen). All dilutions and subsequent culture was performed in Opti-MEM. FHL124 cells were transfected with siRNA at 5 nM for 48 h, after which cells were either lysed for protein analysis by western blot, fixed with 4% formaldehyde for immunocytochemistry or treated (for analysis of DNA strand breaks by alkaline comet assay).

### 2.5 Immunocytochemistry

Coverslips or tissue samples (isolated flat mounted epithelium from cultured lenses) were fixed with 4% v/v formaldehyde (Sigma-Aldrich) in PBS for 30 min followed by 3 washes in PBS for 5 min. Cells or tissue were permeabilised with 0.5% v/v Triton-x-100 (Sigma-Aldrich) for 30 min. Three washes in PBS containing 0.02% w/v BSA and 0.05% v/v IGEPAL (Sigma-Aldrich) were performed before blocking for non-specific binding sites with normal donkey serum (1:50 in 1% w/v BSA in PBS) for 1 h. PARP-1 primary antibody (Cell Signaling Technology, Danvers, MA, USA) was diluted (1:100 in 1% BSA in PBS) and applied overnight at 4 °C. Three further washes in PBS containing 0.02% w/v BSA and 0.05% v/v IGEPAL were performed followed by addition of secondary antibody (alexa488-conjugated donkey anti-rabbit (Invitrogen) diluted 1:100 in 1% BSA in PBS) for 1 h, protected from light at 37 °C in a humidified atmosphere. Coverslips or tissue samples were then counterstained with DAPI (Sigma-Aldrich) and Texas red-x-phalloidin (Invitrogen). A further 3 washes in 0.02% w/v BSA and 0.05% v/v IGEPAL were performed followed by mounting of samples onto glass microscope slides. Samples were viewed with fluorescence microscopy (widefield microscope Zeiss AxioPlan 2ie, Zeiss, Gottingen, Germany) and images captured with a digital camera and AxioVision software (Zeiss). Image quantification was performed using ImageJ 1.45s image analysis software.

To establish cross-sections of the human lens, non-cultured whole human lenses were fixed in 4% v/v formaldehyde (Sigma-Aldrich) in PBS, dehydrated in a graded alcohol series, cleared in xylene and embedded in paraffin. Sections of lens (6 µm) were cut and mounted on glass slides. Sections were deparaffinised in xylene and rehydrated in descending ethanol concentrations and then subjected to antigen retrieval in sodium citrate buffer (10 mM sodium citrate, 0.05% Tween 20, pH 6.0) for 20 min. After three washes in PBS for 5 min, sections were blocked in 10% normal donkey serum in PBS for 1 h and then incubated with PARP-1 primary antibody (Cell Signaling Technology, Danvers, MA, USA) diluted 1:400 in PBS overnight at 4 °C. Following three further washes in PBS, sections were incubated with alexa488-conjugated donkey anti-rabbit (Invitrogen) diluted 1:400 in PBS for 1 h protected from light. Sections were counterstained with DAPI, washed once in PBS and mounted with a coverslip. Samples were viewed with fluorescence microscopy (widefield microscope Zeiss AxioPlan 2ie, Zeiss, Gottingen, Germany) and images captured with a digital camera and AxioVision software (Zeiss, Cambridge, UK).

### 2.6 Western blot analysis

Cell lysates from FHL124 cells were prepared using M-PER buffer (Thermo Scientific, Basingstoke, UK) supplemented with phosphatase and protease inhibitors and 0.5 M EDTA (Thermo Scientific) at 10 μl/ml immediately prior to use. Total protein content was determined by the BCA assay (Pierce, Thermo Scientific) to enable loading of equal amounts of protein per sample onto 10% SDS-polyacrylamide gels. Proteins were transferred to PVDF membrane using a semidry transfer cell. Membranes were blocked with PBS containing 5% w/v non-fat dry milk and 0.1% v/v Tween-20, hybridised with primary antibody against PARP-1 or β-actin (Cell Signaling Technology), followed by incubation with secondary antibody conjugated with horse radish peroxidase (GE Healthcare, Little Chalfont, UK). Proteins were detected using the ECL Plus Western Blotting Detection System (GE Healthcare).

### 2.7 Alkaline comet assay

To assess levels of DNA strand breaks in FHL124 cells, the alkaline comet assay was performed. Briefly, following experimental treatments, cells were washed with ice cold PBS, harvested, counted, re-suspended in PBS containing 10% DMSO and frozen at −80 °C until the alkaline comet assay was performed as previously described [28], [29].

### 2.8 Measurement of cell death using the lactate dehydrogenase (LDH) assay

At experimental end points, culture medium (of FHL124 cells or whole human lenses) was sampled and assayed for LDH content as a measure of cell death using a Cytotoxicity Detection Kit (LDH) (Roche, Mannheim, Germany) following the manufacturer's instructions. The amount of LDH present in culture medium is proportional to the amount of dead or dying cells in a population. Data was analysed as a percentage change of absorbance at 490 nm from untreated FHL124 cells or whole human lenses

### 2.9 Analysis of human lenses for reduction in visual quality

Lenses were imaged using a charge coupled device (CCD) camera (UVP, Cambridge, UK) with Synoptics software (Synoptics, Cambridge, UK) at the experimental start point (day 0) and at 24 h intervals throughout experiments. Brightfield illumination was used, with a black and white grid placed beneath lenses to give an assessment of visual quality and therefore lens clarity. Visual quality was quantified from these images by measuring standard deviation values of grey scale values obtained from the grid beneath the lens. Values of standard deviation of clear lenses are high whereas when the lens becomes more opaque, the grid becomes less defined/homogenous and the standard deviation values decrease. A background for each image was achieved by selecting a region of the grid adjacent to the lens. This region exhibits the greatest standard deviation and best visual quality and thus a decrease was calculated relative to these values. Image analysis was performed with Image-Pro Premier (MediaCybernetics Inc., MD, USA) analysis software.

## 3 Statistical analysis

One way analysis of variance (ANOVA) with posthoc Tukey's test was performed to determine statistical differences between multiple experimental groups (SPSS 16.0, SPSS Inc., IL, USA) and Student's *t*-test to determine statistical differences between two experimental groups (Excel, Microsoft, WA, USA). A *p* value of ≤0.05 was considered significant.

## 4 Results

### 4.1 PARP-1 expression in human lens cells

**Fig. 1:**
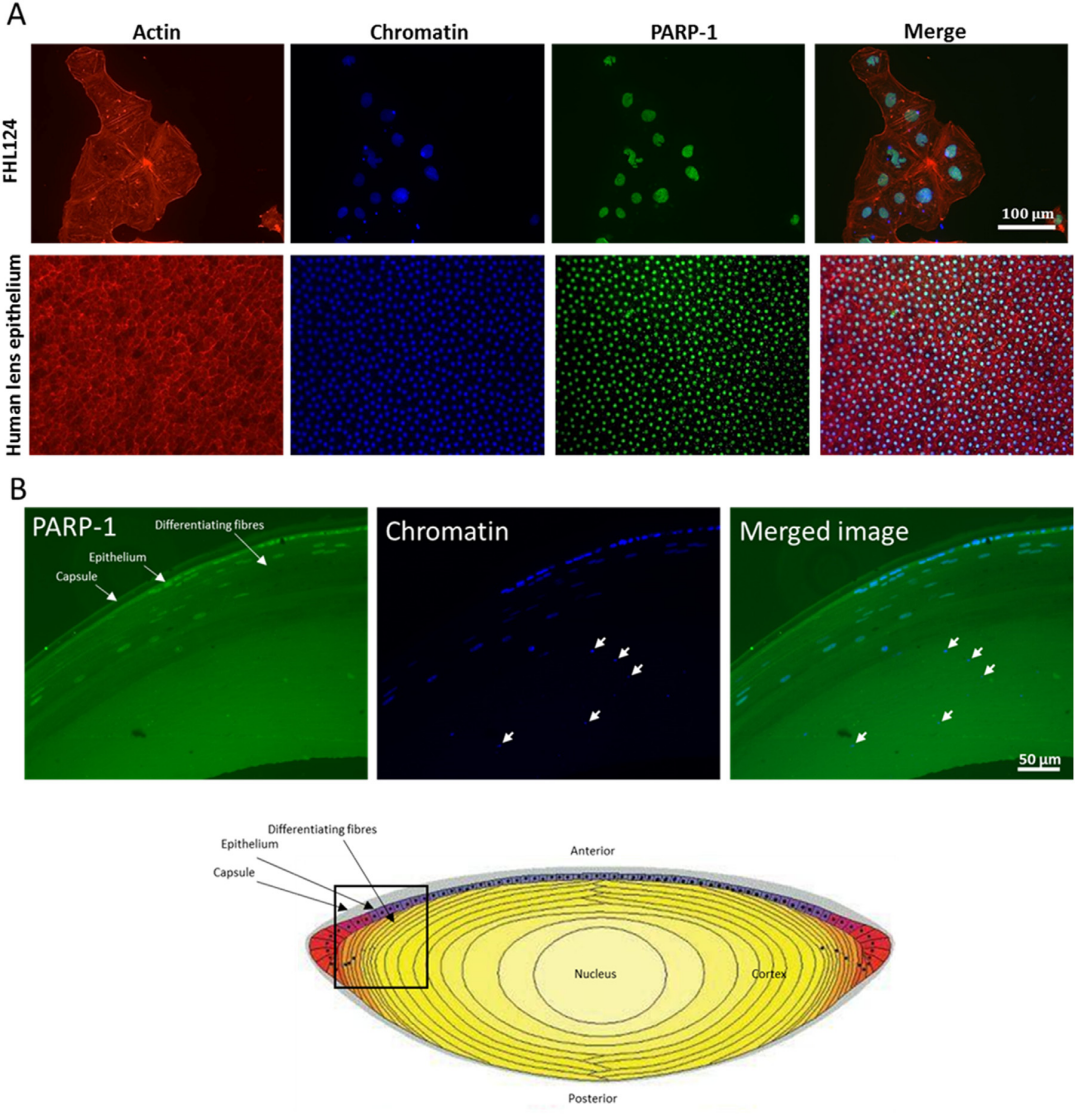
PARP-1 detection in human lens cells. (A) Fluorescent micrographs showing PARP-1 distribution in FHL124 cells and the native human lens epithelium. (B) PARP-1 expression in the lens epithelium and pre-denucleating lens fibre cells. A strong nuclear expression of PARP-1 is observed in cells exhibiting typical nuclei. However, as fibre cells become more embedded in the lens, chromatin appears condensed and these cells do not exhibit PARP-1 (arrowed). The data presented are representative of four lenses from individual donors that were sectioned and stained. As a point of reference a cross-sectional diagram of the lens has been included (adapted from Maidment et al. [44]).

To assess the expression of PARP-1 in FHL124 cells and native human lens immunocytochemistry was performed (Fig. 1A and B). An intense staining pattern for PARP-1 was observed in the nuclear region of both FHL124 cells and the native epithelium (Fig. 1A and B). Newly laid lens fibre cells also presented a predominantly nuclear expression (Fig. 1B), but levels declined at a given point, which seemed to precede changes in chromatin appearance that could be attributed to lens fibre cell de-nucleation (Fig. 1B).

### 4.2 The effects of PARP-1 chemical inhibition on oxidative stress induced DNA strand breaks in FHL124 cells

**Fig. 2:**
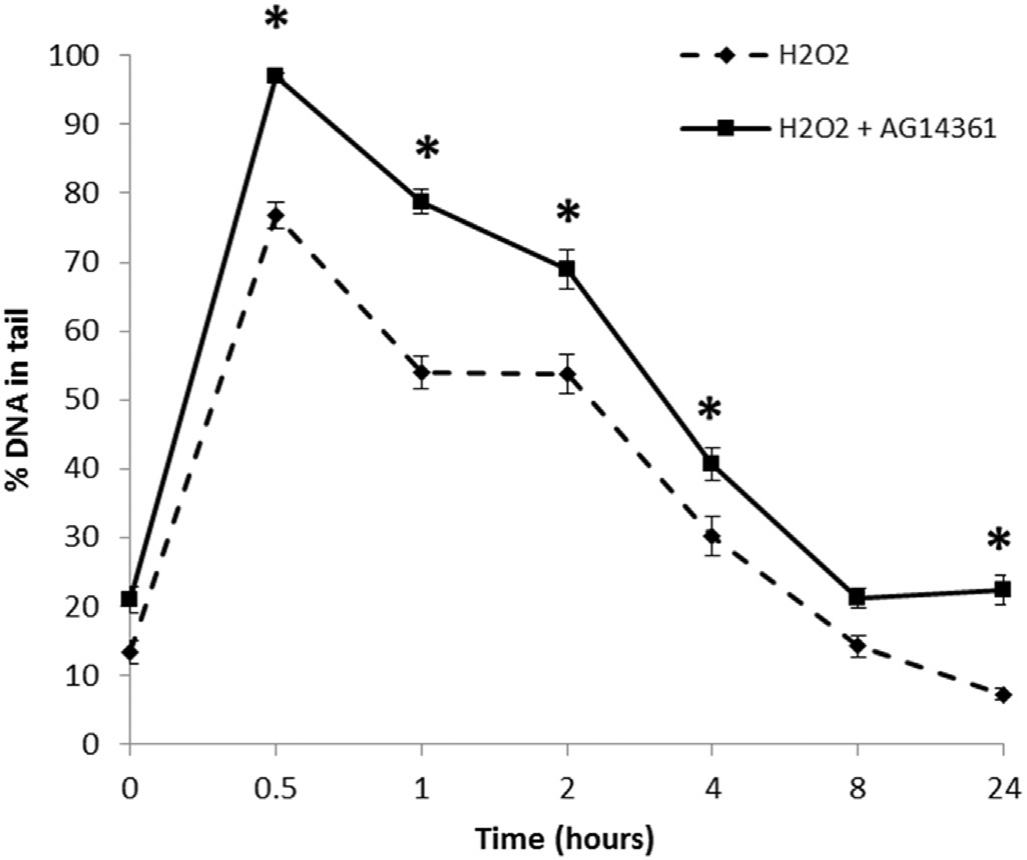
Effect of PARP-1 inhibition on H_2_O_2_-induced DNA strand breaks. FHL124 cells were pre-treated with 1 µM AG14361 for one hour prior to treatment with 30 µM H_2_O_2_. Levels of DNA strand breaks (% DNA in tail) were measured by the alkaline comet assay and compared to H_2_O_2_ treated cells without AG14361 pre-treatment. Data presented is representative of three independent experiments each with 100 comets scored±SEM. *Indicates significant difference between experimental conditions at the indicated time-point (*p*≤0.05; ANOVA with posthoc Tukey's test).

PARP-1 has been clearly described as having important roles in the repair of oxidative DNA damage and single strand break repair via the BER pathway [1], [6], [36]. H_2_O_2_ is a pro-oxidant able to cause oxidative stress and subsequent damage to DNA. To investigate the role of PARP-1 in oxidative stress-induced DNA strand breaks and their repair in human lens cells, a chemical inhibitor of PARP-1, AG14361, was applied at a concentration of 1 µM to FHL124 cells for 1 h prior to treatment with 30 µM H_2_O_2_. DNA strand breaks were measured over 24 h following treatment (Fig. 2). A 1 µM AG14361 concentration applied to human cells has been shown previously to inhibit PARP-1 activity by greater than 90% [34].

H_2_O_2_ treatment in FHL124 cells without PARP-1 inhibition produced a peak level of DNA strand breaks at 0.5 h (76.8±2.0% DNA in tail) following treatment, after which levels of DNA strand breaks were shown to steadily decline over the 24 h studied. In FHL124 cells pre-treated with PARP-1 inhibitor, these levels of DNA strand breaks also peaked at 0.5 post-H_2_O_2_ treatment (97.0±0.5% DNA in tail), but this peak was significantly greater than control cells without PARP-1 inhibition. In fact, for all measurements with PARP-1 inhibited, levels of DNA strand breaks generally remained elevated over the 24 h study period.

### 4.3 The effects of PARP-1 chemical inhibition on oxidative stress-induced FHL124 cell death

To investigate the effects of PARP-1 inhibition on oxidative stress-induced cell death and survival, FHL124 cells were pre-treated with the PARP-1 chemical inhibitor, AG14361, followed by H_2_O_2_ treatment and analysis of cell death and survival. On this occasion FHL124 cells were treated with 0 or 100 µM H_2_O_2_, a concentration chosen based upon previous work within our laboratory that was shown to induce death of these cells [33].

**Fig. 3:**
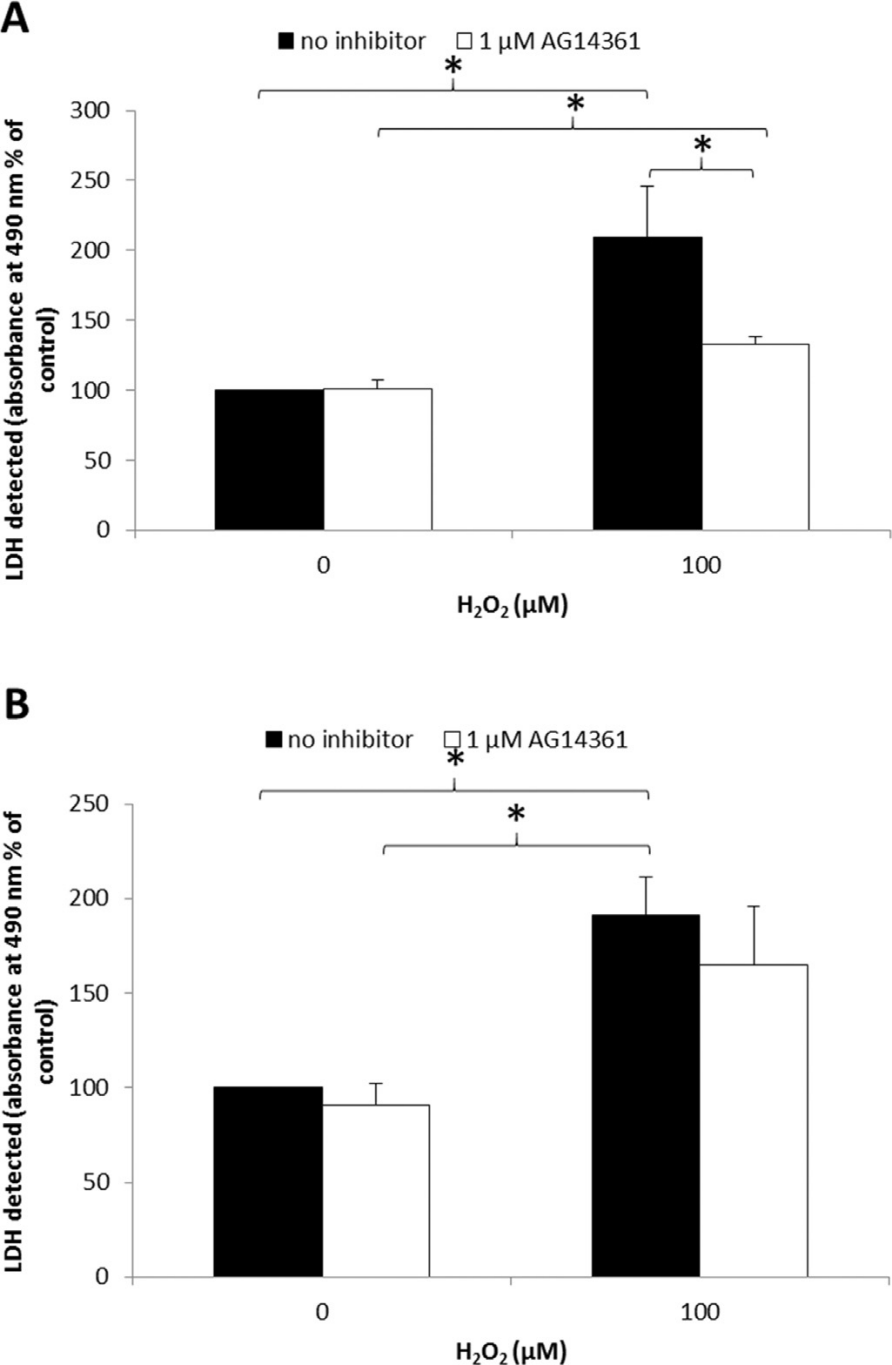
PARP-1 inhibition reduces H_2_O_2_-induced FHL124 cell lysis and death. (A) FHL124 cells were pre-treated with 1 μM AG14361 before application of 100 μM H_2_O_2_. LDH released into the medium was measured (as a marker of cell death) at 24 h (absorbance at 490 nm) and compared to cells without pre-treatment with AG14361. (B) PARP-1 inhibitor (AG14361) is not a direct antioxidant. AG14361 and H_2_O_2_ were added to FHL124 cells simultaneously. LDH release (absorbance at 490 nm) was measured 24 h posttreatment and compared to cells treated with H_2_O_2_ alone. Data represent the mean of three independent experiments±SEM. *Indicates significant difference between experimental groups (*p*≤0.05; ANOVA with posthoc Tukey's test).

Levels of LDH released into the cell culture medium at the experimental end point were assayed as a measure of cell death (Fig. 3). At 24 post-H_2_O_2_ treatment, levels of LDH were significantly lower in FHL124 cells pre-treated with AG14361 compared to those without (Fig. 3A). Levels of LDH were significantly elevated in culture medium of FHL124 cells treated with H_2_O_2_ alone, with a 109.6% increase noted compared to untreated cells. FHL124 cells first pre-treated with AG14361 prior to addition of H_2_O_2_ demonstrated a 32.9% increase in LDH release following H_2_O_2_ treatment compared to untreated cells. No changes in LDH release were noted with inhibitor pre-treatment alone (Fig. 3A).

To address concerns that AG14361 could be directly acting as an anti-oxidant against H_2_O_2_, experiments were undertaken whereby AG14361 and H_2_O_2_ were added to FHL124 cells simultaneously; following 24 h LDH release was again measured (Fig. 3B). No statistically significant difference in LDH release was observed between cells treated with AG14361 and H_2_O_2_ simultaneously and those treated with H_2_O_2_ alone (Fig. 3B).

In summary, analyses of survival and death of FHL124 cells generated consistent findings. For cells treated with H_2_O_2_, pre-treatment with the PARP-1 inhibitor AG14361 improved their survival and reduced levels of cell death.

### 4.4 The effects of siRNA mediated PARP-1 knockdown on oxidative stress-induced DNA strand breaks in FHL124 cells

**Fig. 4:**
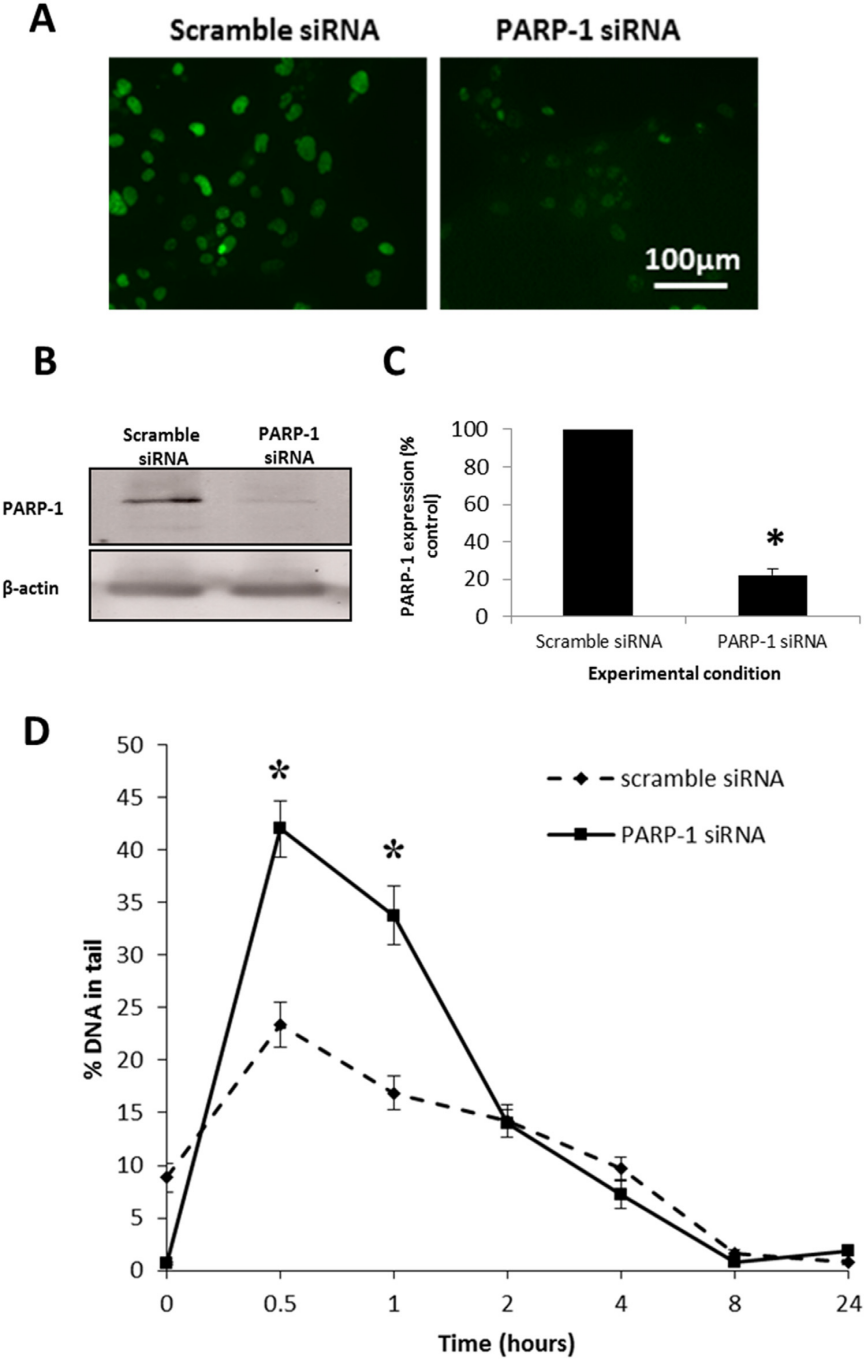
Effect of depleting PARP-1 on H_2_O_2_-induced DNA strand breaks. Targeted siRNA significantly depletes PARP-1 expression in FHL124 cells. (A) Immunocytochemistry demonstrating PARP-1 knockdown, (B) representative gel from western blot and (C) quantification of western blot data pooled from three independent experiments adjusted for β-actin loading controls ± standard error of the mean. *Indicates significant difference (*p*≤0.05; Student's *t*-test). (D) FHL124 cells were treated with siRNA targeted against PARP-1 and treated with 30 µM H_2_O_2_. Levels of DNA strand breaks were measured by alkaline comet assay and compared to levels in FHL124 cells treated with non-coding scramble control siRNA. Data presented is representative of three independent experiments each with 100 comets scored±SEM. *Indicates significant difference between experimental conditions (*p*≤0.05; ANOVA with posthoc Tukey's test).

To further establish the effect of PARP-1 expression on FHL124 cell responses to oxidative stress, PARP-1 was depleted with targeted siRNA. Knockdown of PARP-1 was confirmed by immunocytochemistry and western blotting (Fig. 4A–C). Quantification of PARP-1 western blotting demonstrated that PARP-1 was significantly depleted (77.7±3.3%) compared to cells transfected with non-coding scramble control siRNA. This depletion in PARP-1 expression was in line with expectations provided by the manufacturer.

To investigate the effect of PARP-1 depletion on oxidative stress-induced DNA strand breaks, FHL124 cells were either transfected with PARP-1 siRNA or non-coding scramble control siRNA and treated with 30 µM H_2_O_2_. The alkaline comet assay was performed and levels of DNA strand breaks were measured over 24 h (Fig. 4D). FHL124 cells treated with non-coding scramble siRNA demonstrated a peak level of DNA strand breaks at 0.5 post-H_2_O_2_ treatment (23.4±2.1% DNA in tail) with levels steadily decreasing thereafter. Levels of DNA strand breaks in PARP-1 knockdown cells also peaked at 0.5 post-H_2_O_2_ treatment demonstrating an increase in strand breaks observed in cells without PARP-1 knockdown at the same time point (42.0±2.7% DNA in tail). Importantly, the levels of strand breaks in PARP-1 knockdown cells were significantly elevated compared to cells treated with scramble control siRNA cells at 0.5 and 1 post-H_2_O_2_ treatment (Fig. 4D).

### 4.5 The effect of PARP-1 inhibition on oxidative stress-induced changes in a whole lens culture model

**Fig. 5:**
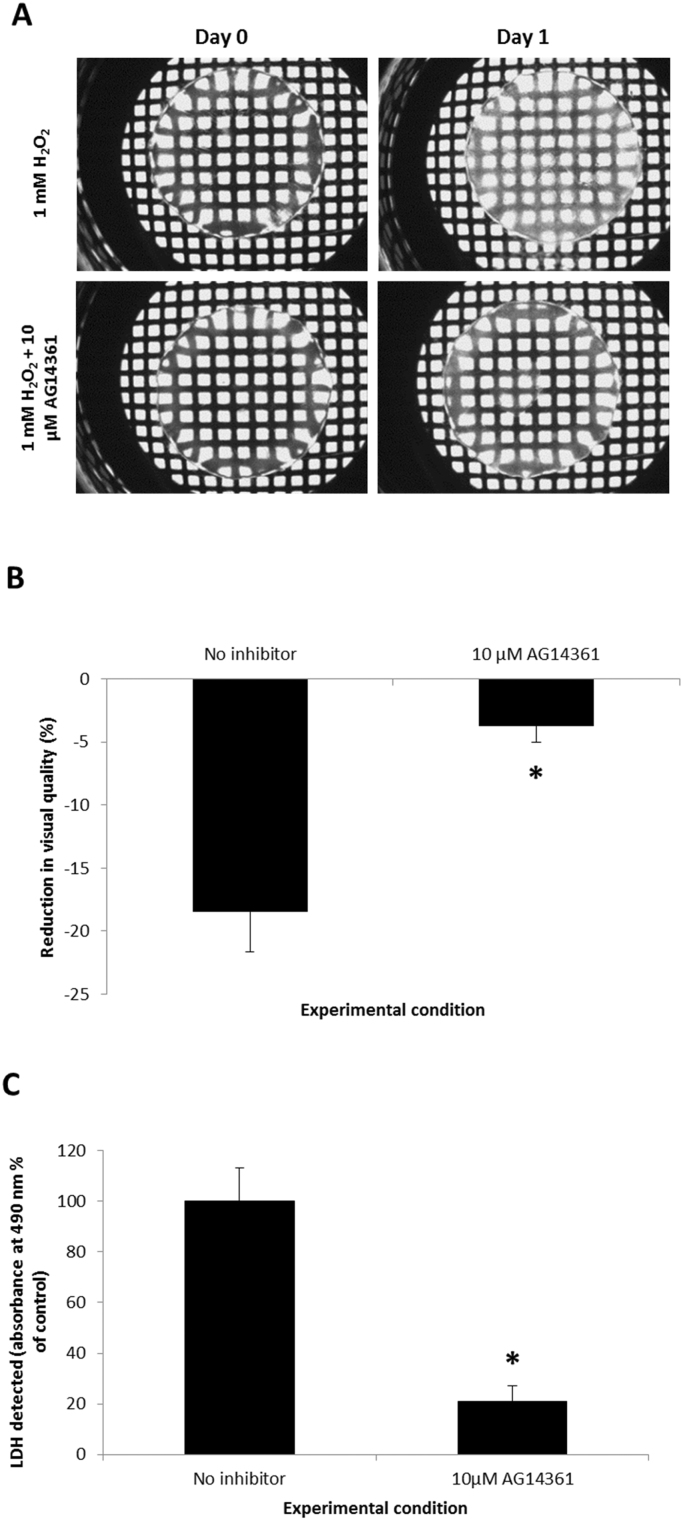
PARP-1 inhibition protects human lenses from oxidative stress induced opacity and cell death. (A) Representative brightfield images of human lenses over time and (B) quantification of visual quality at day 1. (C) LDH release measured at the experimental end point (day 1). Whole human lenses were treated for 1 h with 10 µM AG14361 followed by the application of 1 mM H_2_O_2_ (Day 0) and maintained for a 24 h culture period. Data pooled from three independent experiments±SEM. *Indicates significant differences between conditions (*p*≤0.05; Student's *t*-test).

To test the effect of PARP-1 inhibition on oxidative stress induced changes in the human lens, a whole lens culture system was adopted. Cumulative damage resulting from oxidative stress can occur over a lifetime ultimately giving rise to cataract formation. Our model system applies an acute stress to dramatically induce change to accelerate events and allows us to ascertain whether molecules of interest, in this case a PARP-1 inhibitor, can counter the effects of this insult. A 10 µM solution of AG14361 was applied to the lenses for 1 h prior to treatment with 1 mM H_2_O_2_. A greater concentration of AG14361 was used to inhibit PARP-1 activity in these experiments compared to those using FHL124 cells for various reasons, including increased cell number and potential of the lens capsule to act as a barrier. At 24 h following treatment, lenses were imaged (Fig. 5A) and changes in visual quality were quantified from brightfield images and compared to match paired controls treated with H_2_O_2_ but without pre-treatment with AG14361 (Fig. 5B). Culture medium was also sampled and assayed for LDH release at 24 h as a marker of cell death (Fig. 5C).

At the experimental start point (day 0) lenses in both conditions (1 mM H_2_O_2_ and 1 mM H_2_O_2_+10 µM AG14361) were fundamentally clear. This is demonstrated by the grid beneath the lenses being clearly visible and defined in each instance (Fig. 5A). At day 1, control lenses treated with H_2_O_2_ alone demonstrated a marked decrease in visual quality, with the grid beneath the lens being less visible and defined; however lenses pre-treated with AG14361 were protected from the effects of H_2_O_2_, with the grid remaining visible and only a slight central opacity was noted (Fig. 5A). Quantification demonstrated a significant protection in visual quality compared to match paired controls without pre-treatment with the PARP-1 inhibitor AG14361; 18.4±3.2% versus 3.7±1.4% reduction in visual quality respectively (Fig. 5B).

Pre-treatment with AG14361 also protected lenses from H_2_O_2_-induced cell death. LDH release measured at the experimental end point (day 1) was significantly lower in culture medium sampled from lenses pre-treated with AG14361 compared to lenses without pre-treatment (Fig. 5C), with a 78.9% suppression in LDH release noted.

## 5 Discussion

We have demonstrated that PARP-1 can influence both DNA damage and cell death in human lens cells and, thus, PARPs could putatively affect lens maintenance and disease. The findings of the current study have relevance to maintenance of the healthy human lens and the potential to form lens opacity, and demonstrate a tractable model human cell and tissue system to further investigate links between PARP activity, DNA damage, and the health of cells in general.

Previous work has identified PARP in rodent lens cells [37], [38]. In the present study we have demonstrated PARP-1 expression in the human lens cell line FHL124 and in the native human lens epithelium, and in each instance this expression was predominantly nuclear. For the first time, PARP-1 activity was inhibited in FHL124 lens cells with a specific and potent chemical inhibitor, AG14361 [34], [35] and by a targeted siRNA approach to deplete PARP-1 expression in FHL124 cells. FHL124 cells pre-treated with AG14361 or with knockdown of PARP-1 were found to be more susceptible to oxidative stress-induced DNA strand breaks, with increased sensitivity to H_2_O_2_-induced DNA strand breaks from those treated with H_2_O_2_ alone. This combination of results confirms that effects observed with chemical inhibition of PARP-1 are likely to not be due to a direct anti-oxidant effect, but due to depleted PARP-1 function. Importantly, in both instances repair of DNA strand breaks was eventually observed, which is consistent with previous studies of DNA repair systems using this cell line [28], [29]. In summary, it is evident from these inhibition and knockdown studies that functional PARP-1 has the potential to influence the response to DNA damage in human lens cells, which supports previous findings from a range of studies that have highlighted the importance of PARPs in the response to DNA damage [1], [2], [3], [4], [5], [6]. It can be concluded that PARP-1 inhibition or reduction can render FHL124 cells more susceptible to oxidative stress-induced DNA strand breaks.

The present study has also investigated the effect of PARP-1 inhibition on lens cell survival and death following oxidative insult. Survival of FHL124 cells following H_2_O_2_ treatment was found to be increased when PARP-1 was chemically inhibited, and conversely, this inhibition of PARP-1 afforded protection from oxidative stress-induced cell death. On first observation, this result is in conflict with data suggesting PARP-1 inhibition renders lens cells increasingly sensitive to DNA damage induced by H_2_O_2_. However, PARP enzymes have been reported to play a dual role, such that they can modulate cell death programmes in addition to their well-established pro-survival roles, most notably in DNA repair [23], [39]. The current study has demonstrated that human lens cell death can be mediated by PARP-1. However, the specific pathways in lens cells have yet to be elucidated and this will form the basis of future investigations. In particular, efforts will concentrate on parthanatos whereby PARP-1 is proposed to mediate cell death in response to DNA damage in an alternative pathway to cellular energy depletion. Additional avenues of future investigation will assess the role of PARP-1 in regulating p53 and NF-κB, which are established proteins in cell fate determination [5]. Both are target proteins of PARP-1 and their subcellular location is controlled by the nuclear export protein Crm1; however, their poly(ADP-ribosyl)ation by PARP-1 prevents interaction with Crm1 and their nuclear export. Thus, nuclear accumulation is thought to occur, promoting transcription of genes involved in the stress response and apoptosis [40]. Localisation of p53 and NF-κB could be studied under conditions of cellular stress, and effects of inhibition of PARP-1 elucidated.

In the present study it became apparent that oxidative stress-induced cell death could be suppressed by the inhibition of PARP-1. Thus, experiments were undertaken to ensure that AG14361 was not acting as a direct anti-oxidant. When AG14361 and H_2_O_2_ were added simultaneously to cell culture no difference in cell death was noted compared to H_2_O_2_ treatment alone, suggesting pre-incubation with AG14361 was necessary to produce its inhibitory effect. Crystallographic analysis of AG14361 binding to PARP-1 found it to bind to specific sites within its catalytic domain producing a specific and potent inhibitory effect [34]. This concurs with findings by other investigators that PARP inhibitors do not act as direct antioxidants [41].

Ultimately, PARP-1 inhibition in the human lens suppressed oxidative stress induced opacity. We assume that this benefit is afforded through protection of the epithelium, which ultimately supports the fibre cell population. Previous work in the rat has demonstrated differing effects of PARP inhibition on cataractogenesis. Drel et al. [37] have shown that PARP inhibitors offer some protection against cataract formation in streptozotocin-diabetic rats, which is consistent with the findings in this study. In contrast Miki et al. [38] reported an acceleration of cataract formation in MNU treated rats. Differences between these studies and the results presented could be attributed to the species of investigation, the mode of cell/DNA damage and the PARP inhibitors used for each study, which all differ. Further research is needed to confirm the mechanism(s) by which PARPs could influence oxidative stress induced cataract in humans.

While not a primary focus of our study, the distribution pattern of PARP-1 in lens fibre cells revealed some interesting findings that will be investigated further in future studies. The most newly formed fibre cells presented a strong staining pattern in the nucleus, which reflected observations in the epithelial cell population. The expression levels in the nuclei, however, are dramatically reduced several layers towards the centre of the lens. This loss of expression precedes a change in chromatin organisation that could represent residual nucleoli [42] and we believe could be associated with phased lens fibre de-nucleation [30]. It may be hypothesised that a controlled loss of DNA repair systems will promote DNA damage and contribute to this process. Caspase-3 is also implicated in the denucleation process [30] and PARP-1 is an established substrate for this enzyme [43]. A key player in these apoptotic pathways is p53 [30] and, as described earlier, p53 is a target of PARP-1 [5] and thus its functional impact could at some level be governed by PARP-1. Understanding the regulatory molecules that control PARP activity and establishing the targets of PARP-1 could improve our knowledge of lens fibre denucleation. The findings in the current study therefore provide interesting lines of investigation in the future.

In summary, the current study investigated the role of PARP-1 inhibition on oxidative stress-induced changes in lens cells and provides the first indication that PARP-1 inhibition could supress human lens opacity and decline in visual quality resulting from oxidative stress. This inhibition also afforded protection against cell death. This study lays the foundation to further understand the regulatory functions of PARPs in a human cell and organ context.
